# Erianin: A Direct NLRP3 Inhibitor With Remarkable Anti-Inflammatory Activity

**DOI:** 10.3389/fimmu.2021.739953

**Published:** 2021-10-20

**Authors:** Xinyong Zhang, Lei Hu, Shilei Xu, Chao Ye, Aidong Chen

**Affiliations:** ^1^ Department of Neurology, Huaian Hospital Affiliated to Xuzhou Medical University, Huaian, China; ^2^ The Key Laboratory of Targeted Intervention of Clinical Disease, Collaborative Innovation Center of Translational Medicine for Clinical Disease, Nanjing Medical University, Nanjing, China; ^3^ Guiyang Women and Children’s Hospital, Guizhou Medical University, Guiyang, China; ^4^ Department of General Surgery, The Third Affiliated Hospital, Sun Yat-Sen University, Guangzhou, China

**Keywords:** erianin, inflammasome, NLRP3, traditional Chinese medicine, inflammatory disorders

## Abstract

Erianin (Eri) is the extract of *Dendrobium chrysotoxum* Lindl. The NLRP3 inflammasome is a multiprotein complex that plays key roles in a wide variety of chronic inflammation-driven human diseases. Nevertheless, little is known about the protection of Eri against NLRP3 inflammasome-related diseases. In this study, we demonstrated that Eri inhibited NLRP3 inflammasome activation *in vitro* and *in vivo*. Mechanistically, Eri directly interacted with NLRP3, leading to inhibition of NLRP3 inflammasome assembly. Eri associated with the Walker A motif in the NACHT domain and suppressed NLRP3 ATPase activity. In mouse models, Eri had therapeutic effects on peritonitis, gouty arthritis and type 2 diabetes, *via* NLRP3. More importantly, Eri was active *ex vivo* for synovial fluid cells and monocytes from patients with IAV infection and gout. Eri may serve as a potential novel therapeutic compound against NLRP3-driven diseases.

## Introduction

**Figure 1:**
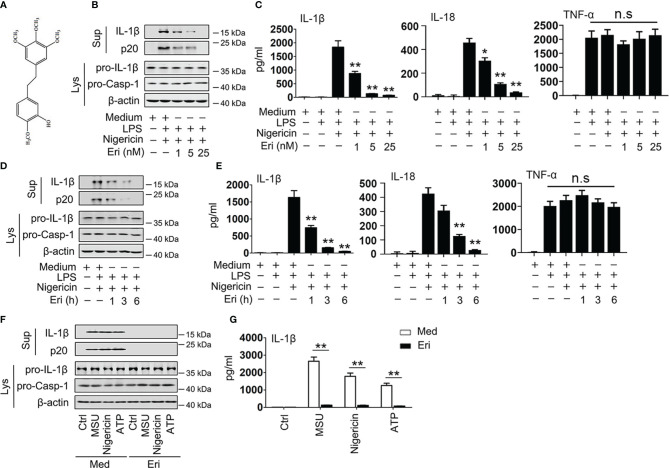
Eri inhibits NLRP3 activation *in vitro.* **(A)** The structure of Eri. **(B)** BMDMs were treated with or without LPS (1 µg/ml) for 3 h, and then treated with nigericin (2 μM) and the indicated concentrations of Eri for 3 h. Mature IL-1β and p20 in supernatants or pro-IL-1β and pro-Casp-1 in lysates were determined by western blot. **(C)** BMDMs were treated with or without LPS (1 µg/ml) for 3 h, and then treated with nigericin (2 μM) and the indicated concentrations of Eri for 3 h. IL-1β, IL-18 and TNF-α protein levels were determined by ELISA. **(D, E)** Experiments were performed as described in **(B, C)**, except that BMDMs were treated with Eri (5 nM) for indicated times. **(F)** BMDMs were treated LPS (1 µg/ml for 3 h), MSU (150 µg/ml for 1 h), nigericin (2 μM for 30 min) or ATP (2.5 mM for 30 min). Then, BMDMs were treated 5 nM Eri for 3 h, mature IL-1β and p20 in supernatants or pro-IL-1β and pro-Casp-1 in lysates were determined by western blot. **(G)** Experiments were performed as described in **(F)**, except that IL-1β protein levels were determined by ELISA. All experiments were repeated at least three times. Bar graphs present means ± SD (**P < 0.01; *P < 0.05). n.s., not significant.

Erianin (Eri) is a natural product isolated from a traditional Chinese medicine, named *Dendrobium chrysotoxum* Lindl (1). Eri is a low molecular-weight chemical compound that possesses two phenyl rings linked by a 2-carbon bridge with several methoxyl substitutions on the phenyl rings (**Figure 1A**) (2). In some ancient Chinese medical books, Eri was reported to has function as an antipyretic and analgesic. In modern times, Eri is used as a chemical marker for the quality control of *D. chrysotoxum*, a type of Dendrobium indexed in the Chinese Pharmacopoeia (2010 version) (3). Recently, several reports found that Eri controlled progression of some diseases, including tumor angiogenesis, diabetic retinopathy and *Staphylococcus aureus* infections (4–6).

Nucleotide and oligomerization domain, leucine-rich repeat-containing proteins (NLRs) are major classes of pattern recognition receptors (PRRs) involved in the innate immune system (7). The NLR family has many members, including NLRP1, NLRC4 and NLRP3, as well as non-NLR receptors, including IFI16 and AIM2 (8). Among those NLR family members, NLRP3 assembles a large multimeric protein complex called inflammasome; this is the best-characterized NLR. NLRP3 protein contains an N-terminal PYRIN domain (PYD), a NACHT-associated domain (NAD), and a C-terminal leucine-rich repeat domain (LRR) (9). Upon activation, the sensor proteins oligomerize, in which ATPase activity of NLRP3 NACHT domain is essential for this process (10). The NACHT domain has two motifs, the Walker A and B motifs. The Walker A motif is necessary for ATP binding, and the B motif is important for ATPase activity (11). After oligomerization, NLRP3 recruits adaptor protein apoptosis-associated speck-like protein containing a CARD domain (ASC), leading to pro-Casp-1 binding to this complex (12, 13). Once assembled, inflammasomes cause auto-cleavage of caspase-1 to generate active subunits p20 and p10 (14, 15). As a result, proinflammatory cytokines IL-1β and IL-18 was released, and pyroptosis, an inflammatory form of cell death, develops (16, 17). Recently, aberrant NLRP3 inflammasome activation has been shown to be related to the progress of several human diseases, including gout, type 2 diabetes (T2D), atherosclerosis and influenza virus- (IV)-induced inflammation (18, 19). Importantly, some traditional Chinese herbs have been reported to exert beneficial effects on NLRP3-related diseases (20, 21). Nevertheless, the unclear pharmacology and complex component of those traditional Chinese herbs limit their clinical application.

Here, we tested whether Eri would alleviate NLRP3 inflammasome activation both *in vitro* and *in vivo*. We then investigated the mechanisms behind this event. On covalently binding to Cys463, Eri blocks NLRP3-NEK7 and NLRP3-ASC interaction and the subsequent NLRP3 inflammasome assembly and activation, leading to an effective suppression of NLRP3-related diseases.

## Methods

### Ethics Statement

Clinical samples collection was conducted according to the principles of the Declaration of Helsinki. Protocols was approved by the Institutional Review Board of Nanjing Medical University in accordance with guidelines for the protection of human subjects. All study participants provided written informed consent for the collection of samples and subsequent analyses.

Mice were bred and used in specific pathogen-free conditions under protocols approved by Nanjing Medical University. All animal experiments were performed in accordance with the National Institutes of Health Guide for the Care and Use of Laboratory Animals.

### Cells and Mice

Human embryonic kidney cells (HEK293T) were purchased from the American Type Culture Collection (ATCC, #CRL-3216) (Manassas, VA, USA). The human monocytic cell line THP-1 was purchased from CBCAS (Cell Bank of the Chinese Academic of Sciences, Shanghai, China). HEK293T cells were cultured in Dulbecco’s modified Eagle’s medium (DMEM) (Gibco, Grand Island, NY, USA) supplemented with 10% fetal bovine serum (FBS) (Gibco, Carlsbad, USA). THP-1 cells were cultured in RPMI-1640 medium (Gibco, Carlsbad, USA), supplemented with 10% FBS. Mouse bone marrow-derived macrophages (BMDMs) were isolated from mice (6-8 weeks old) bone marrow and cultured for 6 to 7 days in DMEM supplemented with 10% FBS and 30% SN from L929 cells. All cells maintained in an incubator at 37°C in a humidified atmosphere of 5% CO_2_. Wild-type C57BL/6 mice were purchased from Hubei Research Center of Laboratory Animals (Wuhan, Hubei, China). C57BL/6 *Nlrp3^-/-^* mice were provided by Nanjing Qingzilan Technology Co. Ltd. (Nanjing, China). Mice were humanely euthanized when they met certain clinical criteria or at the end of the experiments.

### Human Samples

From 2018 to 2019, peripheral blood mononuclear cells (PBMCs) were obtained from ten healthy donors and five patients who were confirmed H1N1 IAV infection. Peripheral blood mononuclear cells (PBMCs) were isolated using standard Ficoll gradient centrifugation from the peripheral blood of either infected subjects or uninfected controls. PBMCs were transfected with plasmid DNA by electroporation with an Amaxa Nucleofector II device according to the manufacturer’s protocol and then resuspended in RPMI 1640 supplemented with penicillin (100 units/ml) and streptomycin (100 mg/ml). Synovial fluid was obtained from five patients with gout with serum uric acid levels >500 μmol/l and knee effusion. The control for synovial fluid collected from five donors who are undergo arthroscopic surgery. All clinical samples were obtained with the assistance of the The Second Affiliated Hospital of Nanjing Medical University.

### Virus, Reagents and Constructs

The recombinant human influenza A virus A/WSN/33 (H1N1) generated by transfecting MDCK cells with the eight-plasmid transfection system to generate influenza A virus. The eight-plasmid transfection system was a gift from R. G. Webster (Department of Infectious Diseases, St. Jude’s Children’s Research Hospital, Memphis, TN, US). The stock virus was propagated in 10-day-old embryonated chicken eggs (Shijun Li laboratory at Central China Agricultural University) for 36-48 h at 37°C. Allantoic fluid was then harvested, and aliquots were stored at -80°C before use. Nigericin, MSU, ATP and poly(dA:dT), and BAY 11-7082 were purchased from SigmaAldrich. SiRNA-NLRP3 (siG000114548A-1-5 and siG000114548B-1-5) and negative control (siRNA-control) (siN0000001-1-5) were purchased from Ruibo Corporation (Guang Zhou, China). Efficiency of RNA interferences was determined using western blotting analyses (**Figure S6D**). The coding regions of NLRP3, MEK7, ASC and Casp-1 were created in our laboratory. The details of antibodies are listed in **Table S1**. To verify constructs and the specificity of antibodies, all constructs were transfected into 293T cells, and expression was analyzed using western blot. All constructs were confirmed by DNA sequencing (Sangon Biotech, Shanghai, China).

### High Fat Diet and Eri Treatment

Six-week-old WT or *Nlrp3^-/-^* mice with similar body weights and plasma glucose levels were randomized into different groups. For generation of HFD-induced diabetic mice, mice were fed with HFD for 14 weeks. The diabetic mice were treated with Eri by intraperitoneal injection once a day for 6 weeks. The mice were maintained with HFD when used for Eri treatment and the subsequent experiments.

### Blood Glucose Assay

Glucose levels in blood collected from the tail vein were determined using a OneTouch Ultra Blood Glucose Test System kit (Roche).

### Human NLRP3 Homology Modeling and Docking Calculation

The human NLRP3 model was prepared by ModWeb Server, which uses the MODELLER program in homology modeling. One chain from crystal structure of rabbit NOD2 bound with ADP (Protein Data Bank accession no. 5IRM) was used as template structure. Small-molecule docking was done by AutoDock with AutoDockTools. The model was defined as rigid, while the ligand was flexible. The model with the best conformation of ligand was further optimized by molecular dynamics simulation using Gromacs. After that, docking was repeated once again. Figures were generated with PyMol (http://www.pymol.org).

### MSU-Induced Peritonitis and Gouty Arthritis

Six-eight-weeks-old C57BL/6 mice were used to induce peritonitis by intraperitoneal administration of 1 mg MSU crystals (dissolved in 0.5 ml PBS). Before injection of MSU, Eri (5 mg/kg) were injected intraperitoneally. After 12 h, the mice were killed and 10 ml ice-cold PBS were used to wash the peritoneal cavities. The polymorphonuclear neutrophils in peritoneal lavage fluid was analyzed by flow cytometry by staining Ly6G and CD11b. The cytokines level in serum or peritoneal lavage fluid was determined by ELISA. For inducing joint inflammation, mice were administered Eri by intraarticular injection. After 1 h, 0.5 mg MSU (dissolved in 20 μL PBS) was administrated intraarticularly and then the size of joints was measured at different time points. After 24 h, the patella were isolated and cultured in 200 μl opti-MEM medium containing 1% Penicillin-Streptomycin for 1 h.

### Western Blot and Co-Immunoprecipitation Analysis

Total cell lysates were obtained, using a Triton X-100 lysis buffer (20 mM Tris (pH 7.5), 150 mM NaCl, 1 mM EDTA, 1 mM EGTA, 1% Triton X-100, 2.5 mM Sodium pyrophosphate, 1 mM β-glycerophosphate, 1 mM Na_3_VO_4_), supplemented with a protease inhibitor-cocktail (Roche applied science) and PMSF (1mM). Protein concentration was determined by Bradford assay (Bio-Rad, Hercules, CA, USA). Protein samples (50 μg) were resolved in SDS-PAGE and were transferred to PVDF membranes (Millipore, MA, USA). PVDF membranes were blocked with 5% skim milk in PBS with 0.1% Tween 20 (PBST) before being incubated with the antibody. Protein band were detected using a Luminescent image Analyzer (Fujifilm LAS-4000).

For immunoprecipitation assays, 500 μg of cellular extracts were incubated with appropriate primary antibodies or normal rabbit/mouse immunoglobin G (IgG) on a rotator by overnight incubation at 4°C, followed by addition of protein A/G Sepharose CL-4B beads at 4°C. Beads were then washed four times with lysis buffer (50 mM Tris-HCl, pH 7.4, 150 mM NaCl, 1 mM EDTA, 1% NP-40, 0.25% sodium deoxycholate and protease inhibitor mixture). The immune complexes were subjected to Western blot.

### Enzyme-Linked Immunosorbent Assay (ELISA)

The concentrations of cytokines in culture supernatants were measured by the ELISA Kit (BD Biosciences, San Jose, CA, USA) according to the manufacturer’s instructions.

### Protein Purification

For *in vitro* pull-down assays, NLRP3 were cloned into the expression vector pGEX-4T-1 (Amersham Pharmacia, Buckinghamshire, UK) and were expressed in Rosetta (DE3) pLys (Novagen) E. coli cells. The recombinant proteins were further purified on glutathione-sepharose bead (Pharmacia, Buckinghamshire, UK) columns to obtain relatively pure GST-NLRP3. His-NEK7 and His-ASC were induced in the same manner and purified on the Ni-NTA columns (Qiagen, Germany). For the *in vitro* pull-down assays, GST-NLRP3, His-NEK7 and His-ASC were incubated together in various combinations. After short incubation, the reaction systems were immunoprecipitated using agarose-immobilized indicated antibodies or protein A/G agarose beads and were then analyzed by western blotting.

For biotin-Eri pull down assays, magic Dynabeads MyOne Streptavidin T1 was preincubated with free biotin or biotin-labeled Eri in PBS for 1 hour at RT, and then incubated with cell lysates overnight with rotating at 4°C. The beads were washed 3-4 times and analyzed by immunoblotting.

### SDD-AGE

Cells were lysed with Triton X-100 lysis buffer (0.5% Triton X-100, 10% glycerol, 50 mM Tris-HCl, 150 mM NaCL, 1 mM PMSF, and 1×cocktail). Then, lysate resuspended in 1× sample buffer (10% glycerol, 0.5× TBE, 2% SDS and 0.0025% bromophenol blue) and loaded onto a vertical 1.5% agarose gel. After electrophoresis in the running buffer (1× TBE and 0.1% SDS) for 1 h with a constant voltage of 80 V at 4°C, the proteins were transferred to Immobilon membrane (Millipore) for immunoblotting.

### MST Assay

A range of concentrations of Eri (0.025 mM-1.2 nM) were incubated with 200 nM of purified GST-NLRP3 protein for 40 min in assay buffer (100 mM NaCl, pH 7.5; 50 mM HEPES; 0.05% Tween 20 and10 mM MgCl_2_). The samples were loaded into the NanoTemper glass capillaries, and MST was performed using 80% MST power and 100% LED power. NanoTemper software in the Monolith NT.115 instrument (NanoTemper Technologies) was use to measure the *K_D_* value. The *K_D_* value was calculated using the mass action equation from duplicate reads of an experiment.

### NLRP3 ATPase Activity and ATP Binding Assay

For ATPase activity assay, purified recombinant human proteins (1.4 ng/µl) were incubated with Eri for 15 min in the reaction buffer at 37°C. Then, ATP (25 µm) was added and incubated for 40 min at 37°C. The amount of ATP converted into adenosine diphosphate (ADP) was determined by luminescent ADP detection with ADP-Glo Kinase Assay kit (Promega, Madison, MI, USA) according to the manufacturer’s protocol. The results were expressed as percentage of residual enzyme activity to the vehicle-treated enzyme.

For ATP binding assay, purified NLRP3 protein (0.1 ng/µl) were incubated with ATP binding agarose for 1 h and then Eri was added and incubated for 2 h with motion at 4°C. Beads were washed and boiled in loading buffer. Samples were subjected to immunoblotting analysis

### Statistical Analysis

Data were obtained from three independent reproducible experiments. Data were expressed as mean ± standard deviations (SD) or mean ± the standard error of the mean (SEM). Student’s t-test was used for statistical comparisons between two groups. One-way analysis of variance (ANOVA) was used to compare three or more groups. A p-value < 0.05 was considered significant and was indicated with an asterisk (*).

## Results

### Eri Inhibits NLRP3 Inflammasome Activation *In Vitro*

Erianin (Eri) has a special chemical structure (**Figure 1A**) that has been previously demonstrated to inhibit tumor angiogenesis (6). The assembly of NLRP3 inflammasome results in the activation of caspase-1, which promotes the cleavage of pro-IL-1β and pro-IL-18 to produce mature and functional IL-1β and IL-18 (22). To test whether Eri is an inhibitor of the NLRP3 inflammasome, we assessed the role of the Eri on caspase-1 activation, IL-1β and IL-18 secretion. As shown in **Figures 1B, C**, Eri inhibited caspase-1 cleavage, IL-1β and IL-18 secretion in a dose-dependent manner in bone marrow-derived macrophages (BMDMs), but had no effects on inflammasome-independent TNF-α production. Similarly, Eri also exhibited time-dependent inhibitory effects on caspase-1 cleavage, IL-1β and IL-18 secretion (**Figures 1D, E**). Interestingly, Eri exhibited inhibitory effects on caspase-1 cleavage and IL-1β secretion was stimulated by other NLRP3 agonists, including ATP and monosodium urate crystals (MSU) (**Figures 1F, G**). By contrast, Eri had no effect on AIM2 and NLRC4 inflammasome activation, which was induced by poly A:T transfection and *Salmonella* infection, respectively (**Figures S1A–D**). Eri had no effect on LPS-induced TNF-α production, pro-IL-1β, or NLRP3 expression (**Figures S1E, F**). A previous study showed that LPS-induced priming does not induce NLRP3 ubiquitination (23). Consistent with this result, we found that Eri did not affect the ubiquitination of NLRP3 during LPS-induced priming (**Figure S1G**). Taken together, these data suggest that Eri is a specific inhibitor for NLRP3 activation *in vitro*.

### Eri Inhibits NLRP3 Inflammasome Activation *In Vivo*

**Figure 2:**
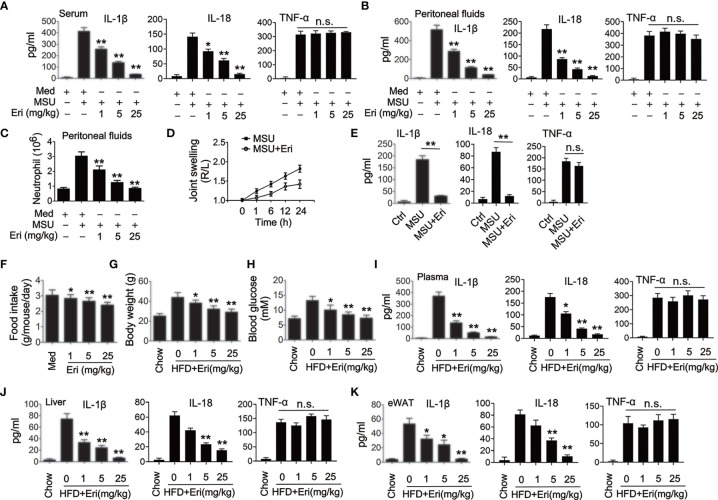
Eri inhibits NLRP3 activation *in vivo.* **(A, B)** C57BL/6 mice were intraperitoneally injected with the indicated concentrations of Eri for 12 h. Then, mice were intraperitoneally injected with MSU (1 mg/mouse) for 6 h. IL-1β, IL-18 and TNF-α protein levels in serum **(A)** or peritoneal fluids **(B)** were determined by ELISA. **(C)** Experiments were performed as described in **(B)**, except neutrophil numbers in the peritoneal cavity were analyzed using FACS. **(D)** C57BL/6 mice were intraarticularly injected with Eri (0.5 mg/kg) for 1 h. Then, mice were intraperitoneally injected with MSU (0.5 mg/mouse). Joint swelling was measured at the indicated time. **(E)** C57BL/6 mice were intraarticularly injected with Eri (0.5 mg/kg) for 1 h.Then, mice were intraperitoneally injected with MSU (0.5 mg/mouse) for 24 h. IL-1β, IL-18 and TNF-α protein levels in joint culture were determined by ELISA. **(F–H)** C57BL/6 mice were fed with HFD for 12 weeks. Then, the mice were treated with the indicated concentrations of Eri every other day for 4 weeks. Food intake **(F)** and body weights **(G)** were recorded and blood glucose levels **(H)** were measured. **(I)** Experiments were performed as described in **(F)**, except IL-1β, IL-18 and TNF-α protein levels in plasma were determined by ELISA. **(J, K)** C57BL/6 mice were fed with HFD for 12 weeks. Then, the mice were treated with the indicated concentrations of Eri every other day for 4 weeks. Liver **(J)** and white adipose tissue (WAT) **(K)** were isolated and cultured for 24 h. IL-1β, IL-18 and TNF-α protein levels in supernatants were determined by ELISA. Bar graphs present means ± SEM, n = 5 for each group (**P < 0.01; *P < 0.05). n.s., not significant.

A previous examined the pharmacokinetic profile of Eri *in vivo* (24), in which the efficacy of Eri was evaluated in mice. Delivery of MSU into the abdominal cavity and the joints resulted in NLRP3-dependent IL-1β production and neutrophil influx and NLRP3-dependent arthritis, respectively (18). We then determined whether Eri suppresses MSU-regulated NLRP3 inflammasome activation *in vivo*. As shown in **Figures 2A–C**, Eri exhibited dose-dependent inhibitory effects on MSU-induced IL-1β, IL-18 production and neutrophils migration in mice. In addition, Eri treatment had no effect on MSU-induced TNF-α production (**Figures 2A, B**). Similarly, Eri treatment also inhibited MSU induced joint swelling, IL-1β and IL-18 production in joint tissue (**Figures 2D, E**). Previous studies showed that the NLRP3 inflammasome is an important contributor for T2D and influenza virus (IV)-induced cytokine expression (18–20). As expected, Eri treatment decreased food intake and weight gain in mice fed with high-fat diets (HFD) (**Figures 2F, G**). Eri treatment also lowered blood glucose levels in HFD-fed mice (**Figure 2H**). As expected, IL-1β and IL-18 production in serum, liver, and adipose tissues of HFD-treated mice was inhibited by Eri treatment (**Figures 2I–K**). Consistent with those results, Eri suppressed IAV-induced IL-1β and IL-18 secretion in a time- and dose- dependent manner in BMDMs and the bronchoalveolar lavage fluid (**Figures S2A–D**). Moreover, we found that Eri had no effects on IAV-induced TNF-α secretion (**Figures S2A–D**). The effects of Eri on the metabolic parameters were investigated in normal lean mice. We found that Eri had no effect on the metabolic parameters and serum chemistry (**Figures S3A–D**). Taken together, these *in vivo* data suggest that Eri may has therapeutic effects on NLRP3 inflammasome related diseases.

### Eri Blocks NLRP3 Inflammasome Assembly

**Figure 3:**
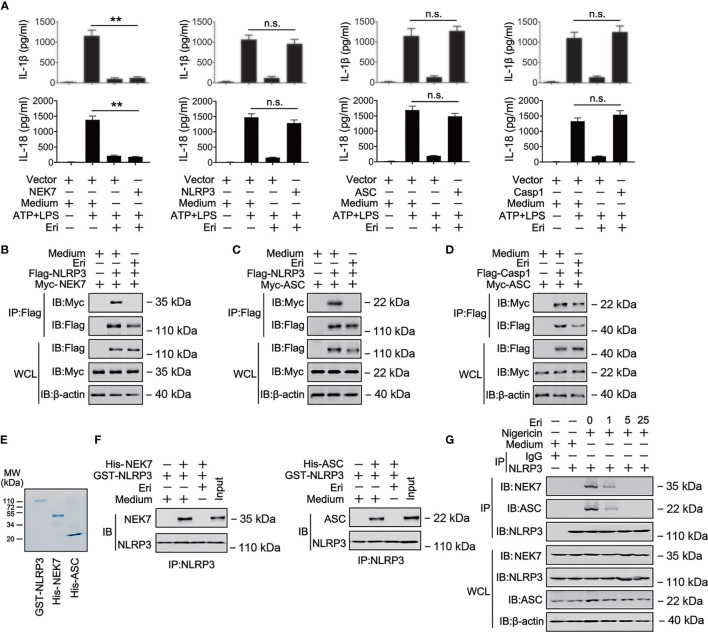
The role of Eri in NLRP3 inflammasome assembly. **(A)** THP-1 cells were co-transfected with the indicated plasmid for 36 h, then treated LPS (1 µg/ml) for 6 h and ATP (2.5 mM for 30 min). IL-1β and IL-18 protein levels were determined by ELISA. **(B)** HEK293T cells were transfected with Flag-tagged NLRP3 (Flag-NLRP3) or Myc-tagged MEK7 (Myc-MEK7) for 36 h. Then, cells were treated with Eri (5 nM) for 6 h. Co-IP and immunoblot analysis were performed with the indicated antibodies. **(C, D)** Experiments were performed as described in **(B)**, except Myc-ASC **(C)** or Myc-ASC and Flag-Casp-1 **(D)** were used. **(E, F)** Purified GST-NLRP3, His-MEK7, and His-ASC were mixed (3 nM) in various combinations as indicated, and incubated with Eri (3 nM). The solution was immunoprecipitated with indicated antibody and analyzed using western blotting. **(G)** THP-1 cells were treated with Nigericin (2 μM for 2 h) and Eri (5 nM for 6 h). Co-IP and immunoblot analysis were performed with the indicated antibodies. All experiments were repeated at least three times. Bar graphs present means ± SD (**P < 0.01; n.s., not significant).

To investigate the mechanism of Eri in NLRP3 activation, we examined the effects of Eri on IL-1β production mediated by various signaling molecules involved in NLRP3 pathways. As shown in **Figure 3A**, Eri-mediated inhibition IL-1β and IL-18 secretion was reversed by overexpression of NLRP3, ASC and Casp-1 but not NEK, suggesting that Eri regulated IL-1β secretion at a step between NEK and NLRP3, or ASC and NLRP3. Co-immunoprecipitation (Co-IP) experiments also suggest that Eri prevents NEK7-NLRP3 and ASC-NLRP3 interaction, but not ASC-Casp-1 interaction (**Figures 3B–D**). The findings were corroborated by *in vitro* binding assays using purified proteins (**Figures 3E, F**). We further performed Co-IP experiments of the endogenous proteins. The results suggested that Eri inhibits NEK-NLRP3 and ASC-NLRP3 interaction in response to nigericin treatment (**Figure 3G**). A previous study found that NEK7 interacted with NEK9 during mitosis (23). However, both exogenous and endogenous Co-IP experiments showed that Eri did not abolish NEK7-NEK9 association (**Figures S4A, B**). Taken together, these data suggest that Eri regulates NLRP3 inflammasome activation by preventing NLRP3 inflammasome formation.

### Eri Is Associated With NLRP3

**Figure 4:**
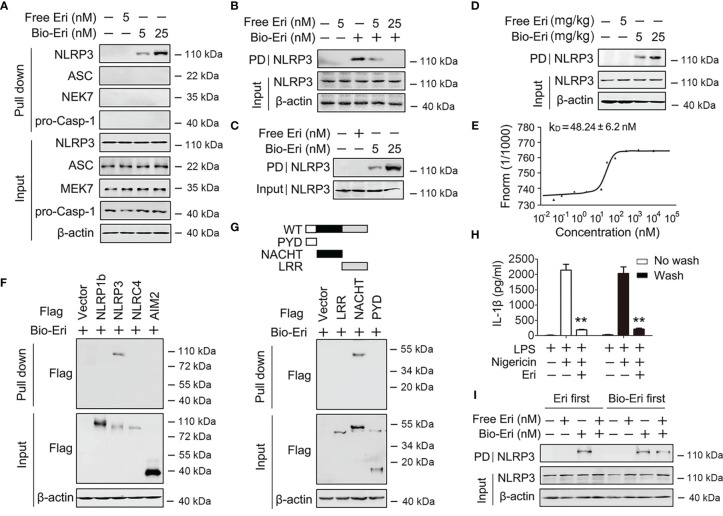
Eri associates with NLRP3. **(A)** Immunoblot analysis of binding complexes isolated from BMDMs extracts incubated with biotin-labeled Eri (Bio-Eri) or free Eri. **(B)** BMDMs extracts were incubated with Bio-Eri and different concentrations of free Ori. The total proteins (input) and pulled down proteins (PD) were immunoblotted as indicated. **(C)** Purified human GST-NLRP3 protein was incubated with indicated doses of Bio-Eri. Total proteins (input) and pulled down proteins (PD) were immunoblotted as indicated. **(D)** C57BL/6 mice were intraperitoneally injected with the indicated concentrations of Eri or Bio-Eri for 12 h. Then, mice were intraperitoneally injected with MSU (1 mg/mouse) for 6 h The total proteins (input) and pulled down proteins (PD) in peritoneal fluids were immunoblotted as indicated. **(E)** MST assay for the affinity between Ori and purified GST-NLRP3 protein. **(F)** HEK293T cells were transfected with indicated plasmids for 36 h. Immunoblot analysis of binding complexes isolated from cells extracts incubated with Bio-Eri. **(G)** Schematic diagram of the full-length and truncated constructs of NLRP3 (upper panel). HEK293T cells were transfected with indicated plasmids for 36 h. Immunoblot analysis of binding complexes isolated from BMDMs hextracts incubated with Bio-Eri (lower panel). **(H)** BMDMs were treated with LPS (1 µg/ml for 6 h) or Eri (5 nM for 15 min), and washed or no washed for three times. Then, cells treated with Nigericin (2 μM) for 2 h. IL-1β protein levels in supernatant were determined by ELISA. **(I)** BMDMs extracts were incubated with free Eri for 4 h before or after incubated with bio-Ori (1 μM) for 4 h. Total proteins (input) and pulled down proteins (PD) were immunoblotted as indicated. All experiments were repeated at least three times. Bar graphs present means ± SD (**P < 0.01).

Because Eri abolished the NEK-NLRP3 and ASC-NLRP3 interactions, we suspected that Eri would directly bind NLRP3. To test this hypothesis, we synthesized biotin-labeled Eri (Bio-Eri) and performed a biotin pull-down assay. As shown in **Figure 4A**, Eri specifically interacted with NLRP3, but not ASC, NEK7 or caspase-1. Interestingly, free Eri competed off Bio-Eri and NLRP3 interaction (**Figure 4B**). The Eri-NLRP3 association was further verified by *in vitro* and *in vivo* binding assays using purified proteins (**Figures 4C, D**). Specifically, using a microscale thermophoresis (MST) assay, we demonstrated that Eri directly interacted with NLRP3, with an equilibrium dissociation constant (K_D_) approximately 50 nM (**Figure 4E**). We also validated the relationship between Eri and other innate immune sensors, including NLRP1B, NLRC4 and AIM2. As shown in **Figure 4F**, Eri specifically interacted with NLRP3, but not NLRP1B, NLRC4 and AIM2. To map the region of NLRP3 that interacted with Eri, we constructed a series of NLRP3 plasmids with Flag-tagged truncation mutants (**Figure 4G**). The biotin pull-down results showed that the NACHT domain of NLRP3 was necessary for its interaction with Eri (**Figure 4G**). We next determined the reversibility of Eri inhibition of NLRP3 inflammasome. As shown in **Figure 4H**, Eri still inhibited nigericin-induced IL-1β release after washout. Moreover, post-treatment of excess amounts of Eri did not prevent Bio-Ori and NLRP3 interaction (**Figure 4I**). Taken together, these data suggest that NLRP3 is an Eri-associated protein.

### Eri Inhibits the Function of NLRP3

**Figure 5:**
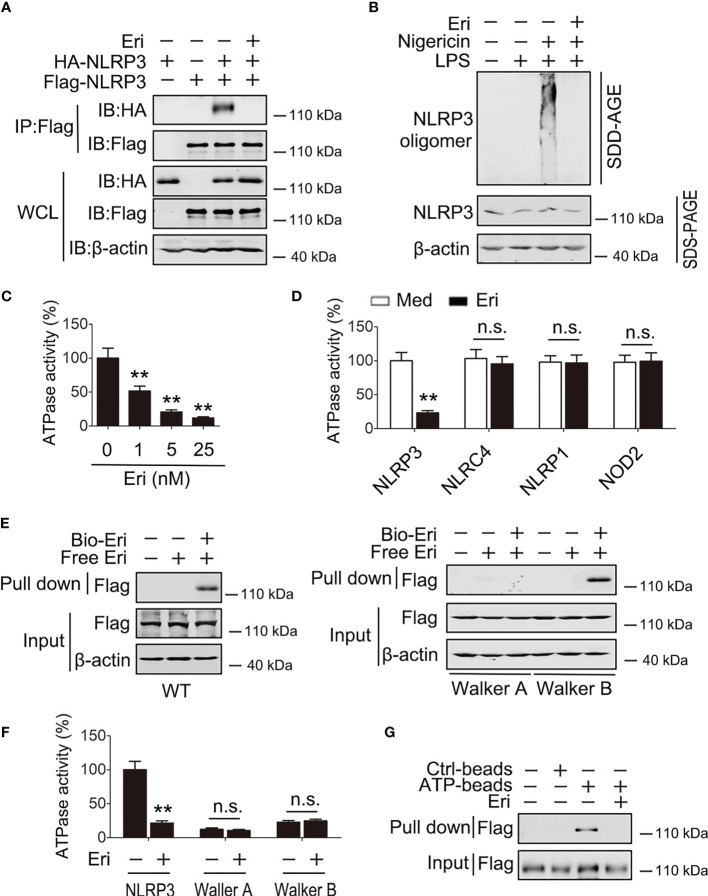
Eri suppresses the ATPase activity of NLRP3. **(A)** HEK293T cells were transfected with indicated plasmids for 36 h. Immunoblot analysis of binding complexes isolated from BMDMs extracts incubated with Bio-Eri. **(B)** BMDMs were treated with LPS (1 µg/ml for 6 h) or Eri (5 nM for 6 h). Cells extracts were analyzed using SDD-AGE (upper panel) and SDS-PAGE (lower panel). **(C)** ATPase activity assay for purified NLRP3 in the presence of different dose of Eri. **(D)** ATPase activity assay for purified NLRP3, NLRC4, NLRP1 and NOD2 with or without presence of Eri (5 nM). **(E)** HEK293T cells were transfected with Flag-NLRP3 (left panel) or NLRP3 constructs with Walker A or Walker B motif mutation (right panel) for 36 h. Immunoblot analysis of binding complexes isolated from cells extracts incubated with Bio-Eri or free Eri. **(F)** ATP-binding assay for purified Flag-NLRP3 in the presence of Eri (5 nM). **(G)** ATPase activity assay for purified Flag-NLRP3 or mutants with or without presence of Eri (5 nM). All experiments were repeated at least three times. Bar graphs present means ± SD (**P < 0.01; n.s., not significant).

Because Eri binds the NACHT domain of NLRP3 and the NACHT domain is essential for NLRP3 oligomerization, we examined the role of Eri on NLRP3 oligomerization. As shown in **Figure 5A**, Eri inhibited the NLRP3-NLRP3 interaction, suggesting that Eri abolished the oligomerization of NLRP3. The effect of Eri on endogenous NLRP3 oligomerization was further confirmed by SDD-AGE (**Figure 5B**). A previous study found that the NLRP3 NACHT domain possesses ATPase activity (10). We therefore tested whether Eri regulated ATPase activity of NLRP3. As shown in **Figure 5C**, Eri suppressed NLRP3 ATPase activity in a dose-dependent manner. Interesting, Eri did not affect ATPase activity of purified NLRC4, NLRP1 or NOD2, suggesting that the inhibitory effect of Eri is specific (**Figure 5D**). The NACHT domain has two motifs, Walker A and B, that are necessary for the ATPase activity of the NACHT domain (10). Although both mutants had no ATPase activity, the interaction of Eri and NACHT domain was inhibited by the mutation of the Walker A motif, but not by mutation of the B motif (**Figures 5E, F**). Consistently, Eri inhibited purified NLRP3 and ATP interactions (**Figure 5G** and **Figure S5A**).

To further evaluate how Eri inhibited NLRP3 ATPase activity, we performed human NLRP3 homology modeling and docking calculation. The results showed that Eri was readily docked into the ATP-binding pocket formed by its NACHT domain (**Figure S5B**). Next, we used BLAST to analyze human NLRP3 NACHT protein sequence and found nine cysteine residues. To determine which cysteine residue was responsible for the binding, we constructed NLRP3 mutants, in which a cysteine was changed to alanine. The results showed that mutation at cysteine 463 (C463A) abolished NLRP3 binding to Eri (**Figure S5C**). In addition, although C463A mutant NLRP3 still bound to NEK7 and ASC, Eri could not block those interactions (**Figures S5D, E**). More importantly, we found that Eri could inhibit the activation of NLRP3 inflammasome in NLRP3-deficient macrophages, which had been reconstituted with WT NLRP3 and C261A mutant NLRP3, but not C463A mutant NLRP3 (**Figure S5F**). TNF-α production was used as negative control (**Figure S5G**). Taken together, these data suggest that Eri may target the ATP-binding site of NLRP3, subsequently inhibiting the oligomerization and ATPase function of NLRP3.

### Eri Prevents NLRP3 Inflammasome Related Diseases *via* NLRP3

**Figure 6:**
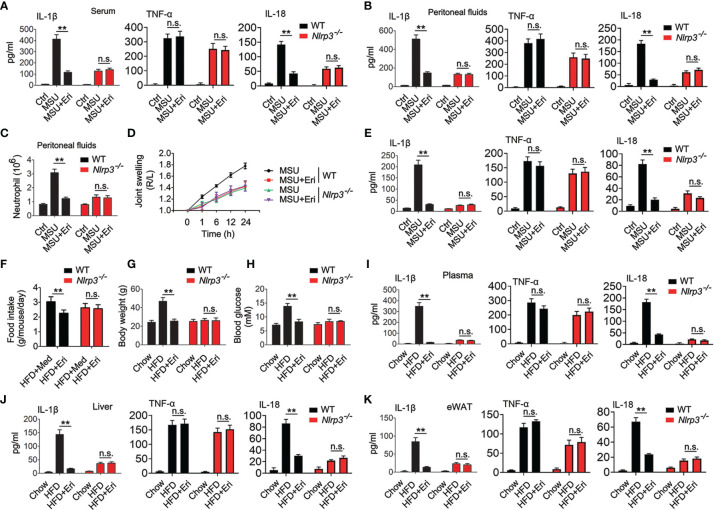
Eri inhibits peritonitis, gouty arthritis and metabolic disorders *via* NLRP3. **(A, B)** WT or *Nlrp3^-/-^* mice were intraperitoneally injected with Eri (5 mg/kg) for 12 h. Then, mice were intraperitoneally injected with MSU (1 mg/mouse) for 6 h. IL-1β, IL-18 and TNF-α protein levels in serum **(A)** or peritoneal fluids **(B)** were determined by ELISA. **(C)** Experiments were performed as described in **(B)**, except neutrophil numbers in the peritoneal cavity were analyzed using FACS. **(D)** WT or *Nlrp3^-/-^* mice were intraarticularly injected with Eri (0.5 mg/kg) for 1 h. Then, mice were intraperitoneally injected with MSU (0.5 mg/mouse). Joint swelling was measured at the indicated times. **(E)** WT or *Nlrp3^-/-^* mice were intraarticularly injected with Eri (0.5 mg/kg) for 1 h. Then, mice were intraperitoneally injected with MSU (0.5 mg/mouse) for 24 h IL-1β, IL-18 and TNF-α protein levels in joint culture were determined by ELISA. **(F–H)** WT or *Nlrp3^-/-^* mice were fed with HFD for 12 weeks. Then, the mice were treated with the indicated concentrations of Eri every other day for 4 weeks. Food intake **(F)** and body weights **(G)** were recorded, and blood glucose levels **(H)** were measured. **(I)** Experiments were performed as described in **(F)**, except IL-1β, IL-18 and TNF-α protein levels in plasma were determined by ELISA. **(J, K)** WT or *Nlrp3^-/-^* mice were fed with HFD for 12 weeks. Then, the mice were treated with the indicated concentrations of Eri every other day for 4 weeks. Liver **(J)** and white adipose tissue (WAT) **(K)** were isolated and cultured for 24 h. IL-1β, IL-18 and TNF-α protein levels in supernatants were determined by ELISA. Bar graphs present means ± SEM, n = 5 for each group (**P < 0.01). n.s., not significant.

To determine whether Eri blocks NLRP3 inflammasome-related diseases *via* NLRP3, we generated mice deficient in the *Nlrp3* gene (*Nlrp3^-/-^* mice) and confirmed the NLRP3 deficiency using semiquantitative RT-PCR and western blot analyses (**Figure S6A**). Eri treatment efficiently inhibited MSU induced IL-1β and IL-18 production and neutrophil influx in WT mice, but not in *Nlrp3^-/-^* mice (**Figures 6A–C**). In addition, Eri treatment had no effect on MSU-induced TNF-α production (**Figures 6A–C**). As expected, Eri also inhibited MSU induced acute joint swelling, IL-1β and IL-18 production in joint tissue of WT mice, but not in *Nlrp3^-/-^* mice (**Figures 6D, E**). The therapeutic effects of Eri on metabolic disorders were also investigated in diabetic mice. As shown in **Figures 6F–H**, less food intake, weight gain and blood glucose were observed in Eri-treated WT mice, but not in *Nlrp3^-/-^* mice. As expected, NLRP3-dependent IL-1β and IL-18 production in serum, liver, and adipose tissues of HFD-treated mice was impaired by Eri treatment (**Figures 6I–K**). Similarly, Eri inhibited IAV-induced IL-1β production in BALF and BMDMs of WT mice, but not in *Nlrp3^-/-^* mice (**Figures S6B, C**). We further examined whether Eri inhibited NLRP3 inflammasome activation *via* NLRP3 *in vitro*. To do this, we designed two specific small interfering RNAs (siRNAs) for NLRP3 and tested their efficiency (**Figure S6D**). Interestingly, the inhibitory effect of Eri on IL-1β production, but not TNF-α, was removed in si-NLRP3 transfected cells (**Figure S6E**). Similarly, Eri also did not inhibit IAV-induced IL-1β production in si-NLRP3 transfected cells (**Figure S6F**). Taken together, those data suggest that is active *in vivo* and can prevent NLRP3-dependent acute inflammation.

### Eri Functions in Cells From Healthy Individuals and Patients

**Figure 7:**
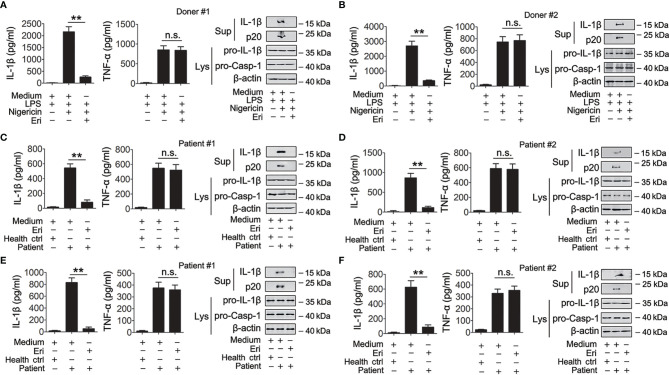
Eri is active for cells from healthy humans, IAV patients or gout patients. **(A, B)** Freshly isolated PBMCs from donor #1 **(A)** and donor #2 **(B)** were treated with or without LPS (1 μg/ml) for 3 h. Then, cells were treated with or without nigericin (2 μM) or Eri (5 nM) for 3 h. IL-1β (left panel) and TNF-α (middle panel) protein levels were determined by ELISA, or mature IL-1β and p20 in supernatants or pro-IL-1β and pro-Casp-1 in lysates were determined by western blot (right panel). **(C, D)** Freshly isolated PBMCs from health donor, IAV patient #1 **(C)** or IAV patient #2 **(D)** were treated with Eri (5 nM) for 6 h. IL-1β (left panel) and TNF- (middle panel) protein levels were determined by ELISA, or mature IL-1β and p20 in supernatants or pro-IL-1β and pro-Casp-1 in lysates were determined by western blot (right panel). **(E, F)** Experiments were performed as described in **(C, D)**, except SFCs from gout patients were used. All experiments were repeated at least three times. Bar graphs present means ± SEM, (**P < 0.01). n.s., not significant.

The role of Eri on NLRP3 inflammasome activation was further evaluated in human primary cells. First, we determined whether Eri was effective in freshly isolated peripheral blood mononuclear cell (PBMC) from five healthy donors. As shown in **Figures 7A, B** and **Figures S7A–C**, nigericin-induced caspase-1 activation and IL-1β production were inhibited by Eri in all five healthy donors. By contrast, Eri had no effect on TNF-α expression (**Figures 7A, B** and **Figures S7A–C**). A previous study found that the NLRP3 inflammasome played an important role in IV-induced IL-1β production (18). Then, we determined whether Eri had an effect on the preactivated NLRP3 inflammasome on PBMCs from five IV patients. As expected, suppressed IL-1β production, but not TNF-α production, was present in Eri-treated PBMCs from all five IV patients (**Figures 7C, D** and **Figures S7D–F**). Similarly, freshly isolated synovial fluid cells (SFCs) from five gout patients were incubated with the presence of Eri, and caspase-1 activation and IL-1β production were inhibited in all patients (**Figures 7E, F** and **Figures S7G–I**). Taken together, these data suggest that Eri or its analogues might be potential drugs, that can be used to control NLRP3-driven diseases.

### The Role of Eri and Other NLRP3 Inhibitors on Inflammasome Activation

A few compounds, including sulforaphane, isoliquiritigenin, BHB, flufenamic acid, mefenamic acid, parthenolide, BAY 11-7082, and MCC950, have been reported to inhibit NLRP3 inflammasome activation (25); we thus compared the activity and specificity of these inhibitors with Eri. Among these inhibitors, Eri and MCC950 showed the best inhibitory activity for NLRP3 inflammasome (**Figure S8A**). Consistent with previous studies (21), sulforaphane and parthenolide could also inhibit AIM2 or NLRC4 inflammasome activation (**Figures S8B, C**), suggesting they are not specific NLRP3 inflammasome inhibitors. Previous studies have shown that sulforaphane, isoliquiritigenin, BHB, flufenamic acid, mefenamic acid, parthenolide, and BAY 11-7082 have inhibitory activity for NF-κB (25). We confirmed these results and found that they could suppress LPS-induced TNF-α production (**Figure S8D**), suggesting that these inhibitors have broad anti-inflammatory activity. In contrast, Eri and MCC950 specifically inhibited NLRP3 inflammasome activation and had no effect on LPS-induced priming effects (**Figures S8A–D**). More importantly, we found that Eri and other NLRP3 inhibitors synergistically inhibited IL-1β production (**Figure S8E**). Together, these results demonstrate that Eri is a specific inhibitor for NLRP3 inflammasome.

## Discussion

Eri is a bibenzyl derivative that is a low molecular-weight natural compound from *Dendrobium chrysotoxum* Lindl (1, 2). It was previously reported that Eri had inhibitory effects on diabetic retinopathy, tumor and retinal angiogenesis (4–6). However, a clear role for Eri in inflammatory responses has not been established. In this study, we showed for the first time that Eri interacts with NLRP3 and exhibited strong anti-inflammasome activity *in vitro* and *in vivo*. These findings suggest that Eri is a potential target for therapy inflammatory disorders.

Inflammasomes are cytosolic multiprotein complexes whose assembly is guided by PRRs (26). To date, five distinct inflammasomes have been identified (27). Among them NLRP3, is the most well-characterized. Several lines of evidence suggest that excessive NLRP3 inflammasome activation is response for several inflammatory disorders, including T2D, atherosclerosis and gout (28). The NLRP3 inflammasome activation is under strict control in the host innate immunity system. Normally, NLRP3 inflammasome activation involves two steps (29). The first is known as the priming signal, controlled by the NF-κB pathway (29). The second step is provided by NLRP3 and its downstream signaling cascades (29). The first step has a robust effect on the inflammatory response, and the second step has a modest effect. Inhibition of either the first or second signal pathway two strategies for treatment of inflammatory disorders. Nevertheless, blockade of the first step may lead to immunosuppression, which increases risks of infections, whereas inhibition of the second step reduces this potential danger. Blocking the activation of NLRP3 inflammasome is therefore a more attractive strategy to interrupt inflammatory and immunological responses. Agents that target IL-1β are currently available treatments for NLRP3-drive diseases (18). However, other inflammasomes or signal pathways also induce IL-1β production (30). For this reason, small-molecule inhibitors such as Eri that target NLRP3 with high specificity may not completely impair host defenses. In this study, we indeed found that Eri had no effect on AIM2 or NLRC4 inflammasomes. These data suggest that Eri might have less immunosuppressive side-effects than blockade of IL-1β. Importantly, our results show that suppression of NLRP3 ATPase activity by Eri has remarkable effects to reduce NLRP3 inflammasome activation. A previous report also showed that CY-09 competes with ATP to bind to NLRP3, and the subsequent reduce NLRP3 inflammasome activation and symptoms in mice models of T2D and CAPS (31). Thus, previous study and our results suggest the ATPase activity could be targeted to screen drug candidates for treatment of NLRP3-drive diseases.

At present, many small molecule inhibitors for NLRP3 inflammasome have been reported and some of them have shown remarkable therapeutic potential. However, none of them is currently approved by food and drug administration (FDA). The unspecific effects of these compounds have limited their clinical potential. These broad-spectrum anti-inflammatory compounds, including BAY 11-7082, sulforaphane, isoliquiritigenin, parthenolide, BHB and INF39, are double-edged swords, important for control of inflammatory responses, but they might also impair host defenses and increase the risk of infection (32). The effects of flufenamic acid and mefenamic acid on chloride efflux indicate that these compounds target the upstream signaling event of NLRP3 and have other unavoidable biological activities (33). A diarylsulfonylurea-containing compound termed as MCC950, is considered one of the most potent and selective inhibitor of NLRP3 inflammasome (34). But the mechanism of MCC950 on NLRP3 inflammasome is not understood. Here we showed that Eri as a specific NLRP3 inflammasome inhibitor that directly targeted NLRP3 itself. In addition, compare with MCC950, Eri has the similar effect to reduce NLRP3 inflammasome activation. More importantly, we found that Eri and other NLRP3 inhibitors synergistically inhibited NLRP3 inflammasome activation (**Figure S8**). The possible reason is that Eri bind a different site of NLRP3. Thus, small-molecule inhibitors with high specificity, such as Eri, might have certain advantages.

Although we reported Eri is a specific inhibitor for NLRP3 inflammasome, some questions remain: (1) Does Eri derivatives have a better therapeutic potential in NLRP3-driven inflammatory diseases? (2) The crystal structure of NLRP3 has not been reported. Why Eri specifically binds with cysteine 463 of NLRP3? (3) If Eri is used on patients, whether it still has anti-inflammatory activity? When considering the next step, studies exploring these questions would be of great help in further clarifying the role of Eri in NLRP3 inflammasome activation.

Overall, our findings demonstrated that Eri selectively suppresses the NLRP3 inflammasome by directly targeting NLRP3. Moreover, both *in vitro* and *in vivo* data showed that Eri is an effective agent for suppression of NLRP3-related diseases. Although Eri clinical application needs optimization of treatment options and integrated safety assessment, Eri may serves as a potential treatment for NLRP3-driven diseases, including IV infection, T2D, atherosclerosis and gout.

## Data Availability Statement

The original contributions presented in the study are included in the article/**Supplementary Material**. Further inquiries can be directed to the corresponding author.

## Ethics Statement

Experiments using animal or human materials (including clinical trials) were approved by the institutional review board of Nanjing Medical University and the institutional animal care and use committee of Nanjing Medical University. The patients/participants provided their written informed consent to participate in this study.

## Author Contributions

XZ participated in carried out all the experiments. SX participated in gene cloning. CY participated in mice experiments. SX collected and analyzed clinical samples for the study. AC participated in the design of the study. All authors contributed to the article and approved the submitted version.

## Funding

This work was supported by the National Natural Science Foundation of China (31571168 and 81970356).

## Conflict of Interest

The authors declare that the research was conducted in the absence of any commercial or financial relationships that could be construed as a potential conflict of interest.

## Publisher’s Note

All claims expressed in this article are solely those of the authors and do not necessarily represent those of their affiliated organizations, or those of the publisher, the editors and the reviewers. Any product that may be evaluated in this article, or claim that may be made by its manufacturer, is not guaranteed or endorsed by the publisher.
