# Supplementary material for: Erianin: A Direct NLRP3 Inhibitor With Remarkable Anti-Inflammatory Activity
Source: Front Immunol. 2021 Oct 20;12:739953. doi: 10.3389/fimmu.2021.739953 (PMC8564113; doi:10.3389/fimmu.2021.739953)
Supplement: Supplementary file 1 [file DataSheet_1.docx]

**Erianin: A direct NLRP3 inhibitor with remarkable anti-inflammatory activity**

**Table of contents**

Supplementary Figures﹒﹒﹒﹒﹒﹒﹒﹒﹒﹒﹒﹒﹒﹒﹒﹒﹒﹒﹒﹒﹒﹒﹒﹒1

Supplementary Materials and Methods﹒﹒﹒﹒﹒﹒﹒﹒﹒﹒﹒﹒﹒﹒﹒﹒﹒﹒6

Supplementary Tables﹒﹒﹒﹒﹒﹒﹒﹒﹒﹒﹒﹒﹒﹒﹒﹒﹒ ﹒﹒﹒﹒﹒﹒﹒9


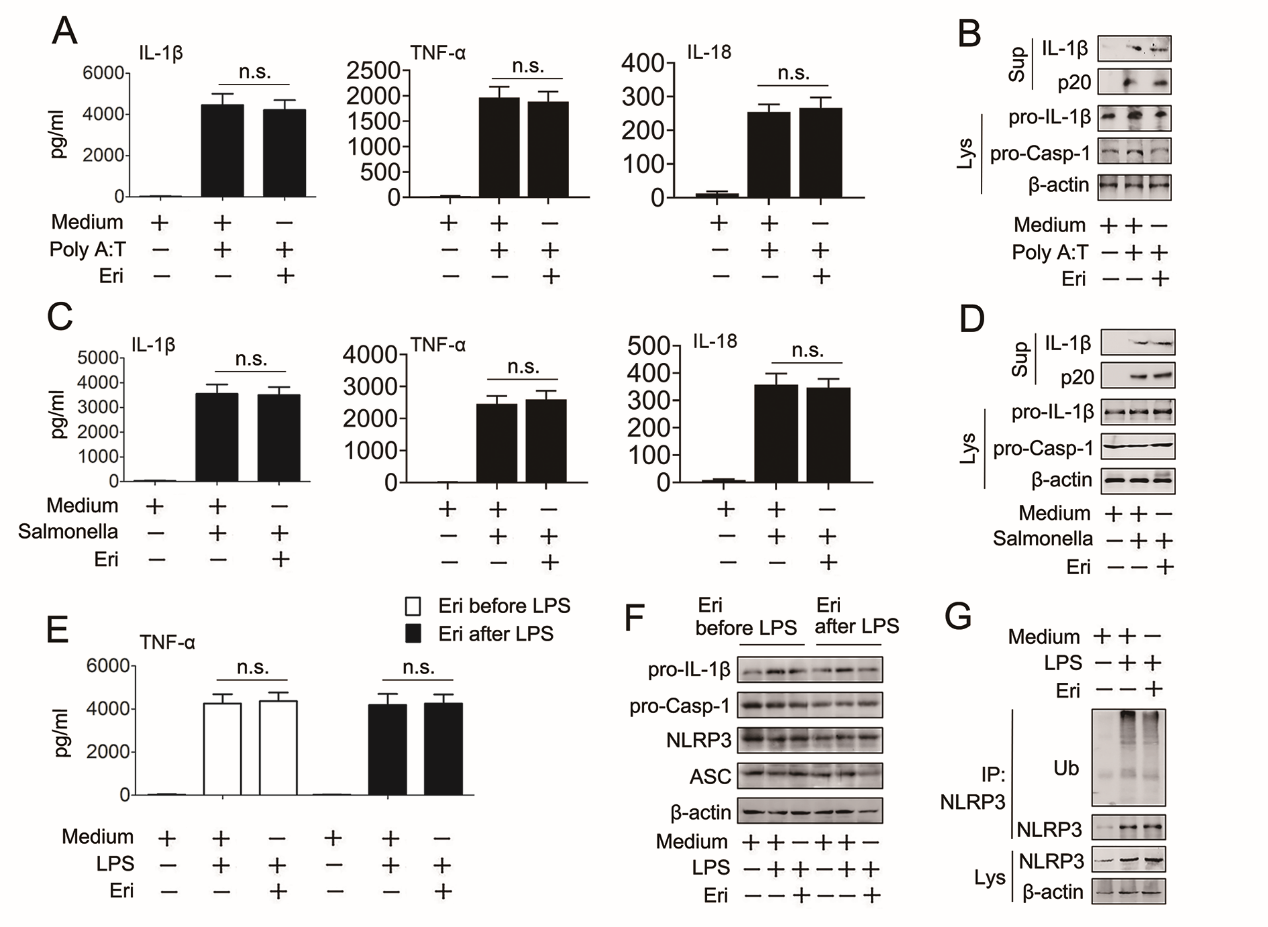


**Supporting Fig. 1. Eri** **has no effects on the activation of NLRC4 or AIM2 inflammasome and LPS-induced priming**

(A) THP-1 cells were treated with Eri (5 nM for 2 h) and then transfected with poly(dA:dT) (0.5 µg/ml for 4 h). IL-1β, IL-18 and TNF-α protein levels were determined by ELISA.

(B) Experiments were performed as described in (A), except mature IL-1β and p20 in supernatants or pro-IL-1β and pro-Casp-1 in lysates were determined by western blot.

(C) THP-1 cells were treated with *Salmonella typhimurium* (multiplicity of infection) or for 30 min and Eri for 6 h. IL-1β, IL-18 and TNF-α protein levels were determined by ELISA.

(D) Experiments were performed as described in (C), except mature IL-1β and p20 in supernatants or pro-IL-1β and pro-Casp-1 in lysates were determined by western blot.

(E) BMDMs were treated LPS (1 µg/ml) for 6 h and then treated with Eri (5 nM) for 3 h, or BMDMs were treated with Eri (5 nM) for 3 h and then treated with LPS (1 µg/ml) for 6 h. TNF-α protein levels were determined by ELISA.

(F) Experiments were performed as described in (E), except western blot assay was performed.

(G) BMDMs were treated with LPS (1 µg/ml) and Eri (5 nM) for 6 h. Co-IP and immunoblot analyses were performed with the indicated antibodies.

All experiments were repeated at least three times. Bar graphs present means ± SD, n = 3 (**P < 0.01; *P < 0.05, n.s., not significant).


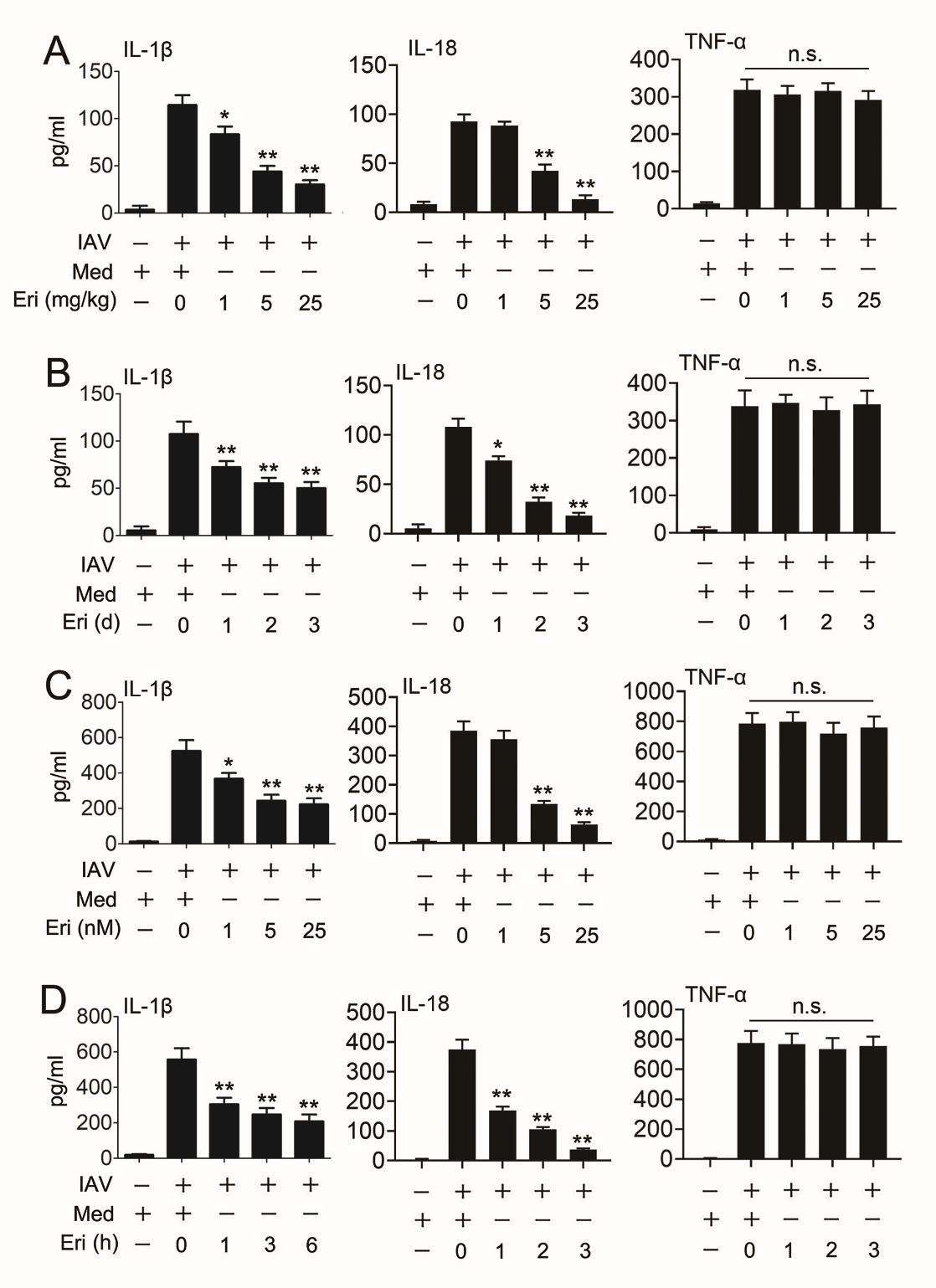


**Supplemental Fig. 2. Eri inhibits IAV-induced** **IL-1β production.**

(A) Mice were intranasally infected with 1×10^4^ pfu of WSN IAV, and were intraperitoneally injected with the indicated concentrations of Eri for 2 d. IL-1β, IL-18 and TNF-α protein levels in the bronchoalveolar lavage fluid (BALF) were determined by ELISA. (n = 3 for each group)

(B) Mice were intranasally infected with 1×10^4^ pfu of WSN IAV, and were intraperitoneally injected with Eri (5 mg/kg) for indicated times. IL-1β, IL-18 and TNF-α protein levels in the bronchoalveolar lavage fluid (BALF) were determined by ELISA (n = 3 for each group).

(C) BMDMs were infected WSN IAV (MOI=1) for 36 h, and treated with the indicated concentrations of Eri for 3 h. IL-1β, IL-18 and TNF-α protein levels were determined by ELISA (n = 3 for each group).

(D) BMDMs were infected WSN IAV (MOI = 1) for 36 h, and treated with Eri (5 nM) for the indicated times. IL-1β, IL-18 and TNF-α protein levels were determined by ELISA (n = 3 for each group).

Bar graphs present means ± SEM, n = 5 (**P < 0.01; *P < 0.05).


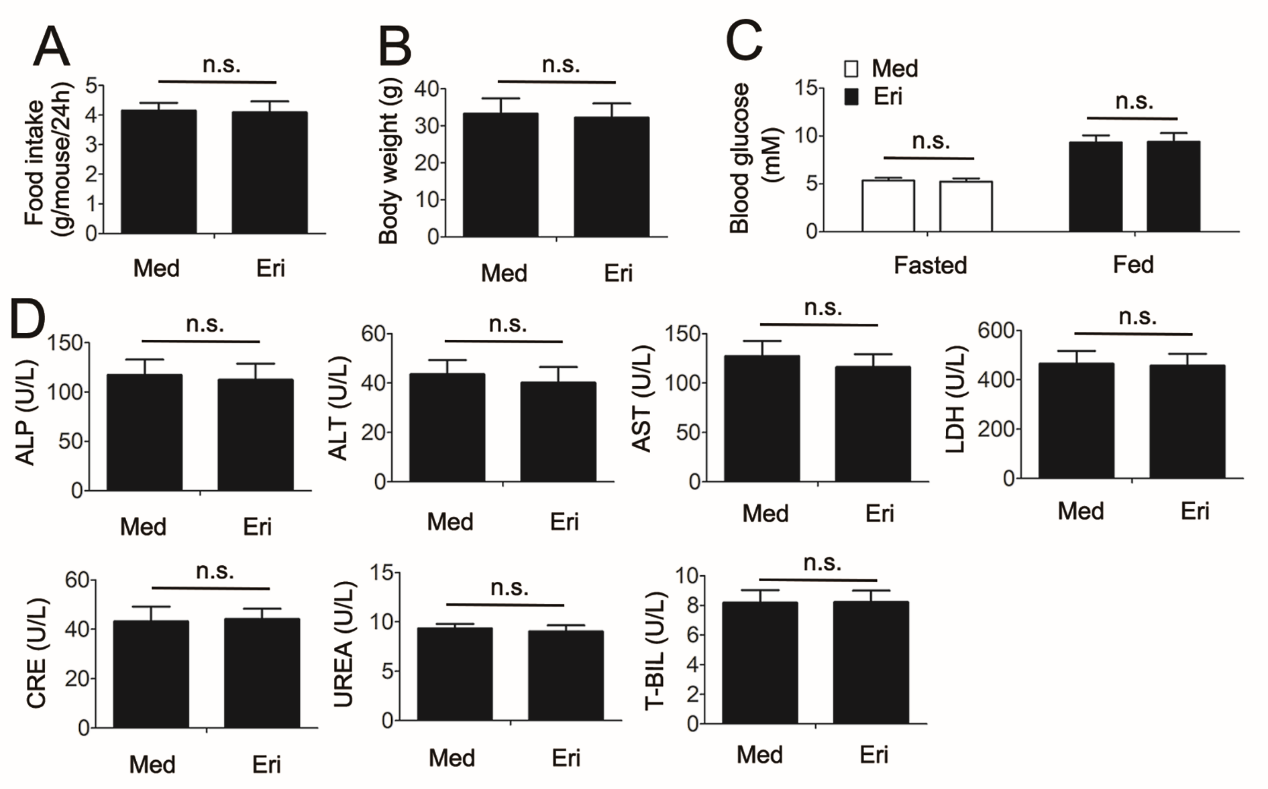


**Supplemental Figure 3. Long-term Eri treatment has no effects on the metabolic parameters and serum chemistry of normal lean mice**

(A-C) C57BL/6J mice treated with Eri (0.5 mg/kg) once a day for 9 weeks. Food intake (A) and body weights (B) were recorded and blood glucose levels (C) were measured.

(D) Qualification of alkaline phosphatase (ALP), alanine aminotransferase (ALT), aspartate aminotransferase (AST), lactate dehydrogenase (LDH), creatinine, urea, and total bilirubin (T-BIL) in the serum of C57BL/6J mice that were treated with Eri (0.5 mg/kg) once a day for 9 weeks.

Bar graphs present means ± SEM, n = 5 (**P < 0.01; *P < 0.05, n.s., not significant).


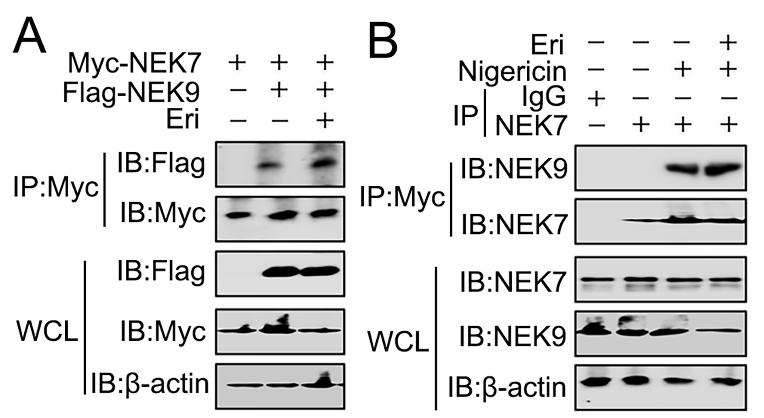


**Supplemental Fig. 4. Eri has no effects on the interaction between NEK7 and NEK9**

(A) HEK293T cells were transfected with Flag-NEK9 or Myc-NEK7 for 36 h. Then, cells were treated with Eri (5 nM) for 6 h. Co-IP and immunoblot analysis were performed with the indicated antibodies.

(B) THP-1 cells were treated with nigericin (2 μM for 2 h) and Eri (5 nM for 6 h). Co-IP and immunoblot analysis were performed with the indicated antibodies.

All experiments were repeated at least three times.


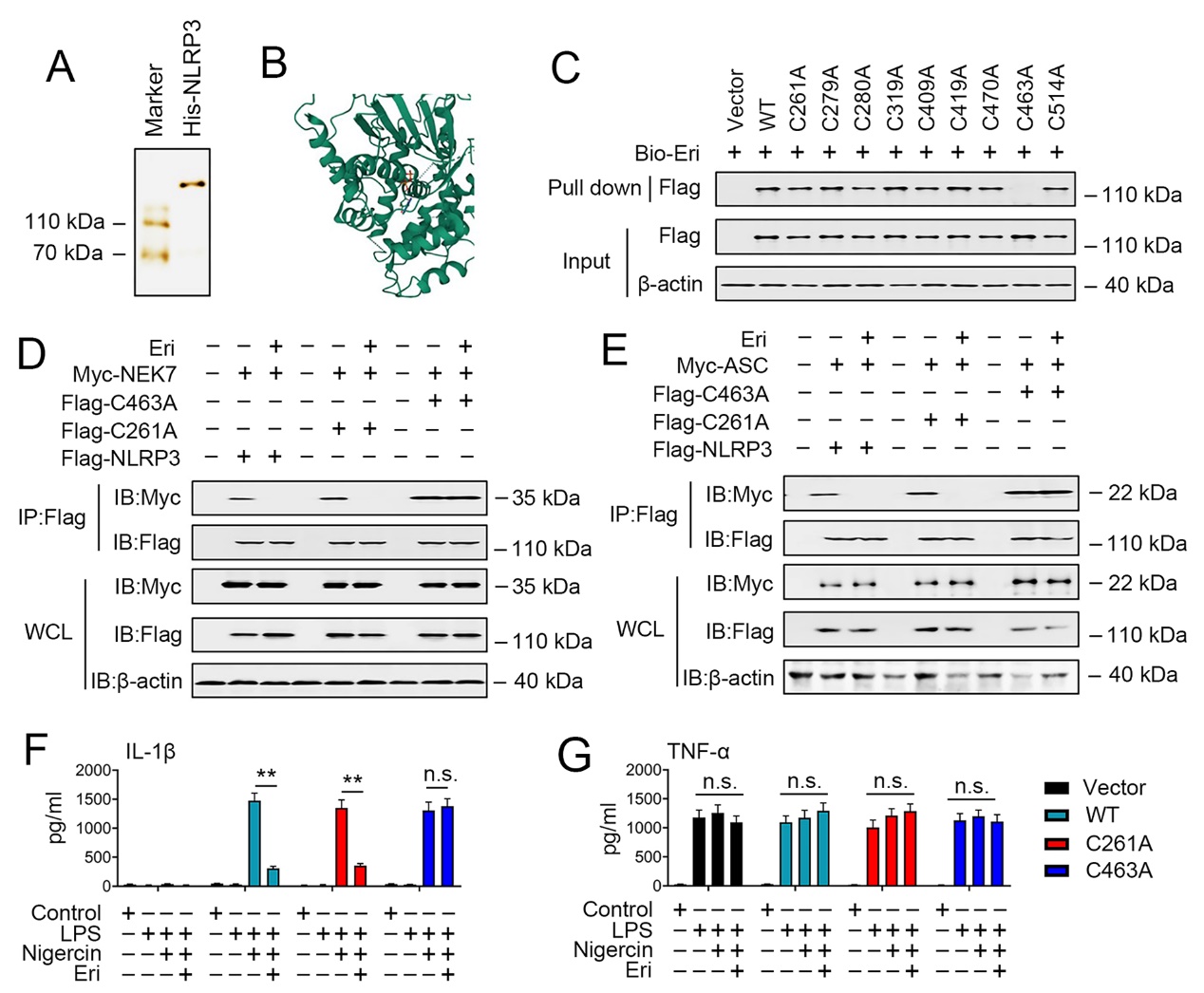


**Supporting Fig. 5. Eri binds to the cysteine 463 on NLRP3.**

(A) Silver staining of the purified His-NLRP3.

(B) Docking complex of NLRP3 with Eri. Eri is shown in sticks and colored red, while NLRP3 is shown in cartoon and colored green.

(C) HEK293T cells were transfected with indicated plasmids for 36 h. Immunoblot analysis of binding complexes isolated from cells extracts incubated with Bio-Eri.

(D) HEK293T cells were transfected with Flag-NLRP3, indicated mutant NLRP3 constructs or Myc-MEK7 for 36 h. Then, cells were treated with Eri (5 nM) for 6 h. Co-IP and immunoblot analysis were performed with the indicated antibodies.

(E) Experiments were performed as described in (d), except Myc-ASC were used.

(F and G) *Nlrp3^-/-^* BMDM reconstituted with WT or indicated mutant NLRP3, then were treated with or without LPS (1 µg/ml for 3 h), and then treated with nigericin (2 μM) and Eri (5 nM) for 3 h. IL-1β and TNF-α protein levels were determined by ELISA.

All experiments were repeated at least three times. Bar graphs present means ± SD (**P < 0.01; *P < 0.05, n.s., not significant).


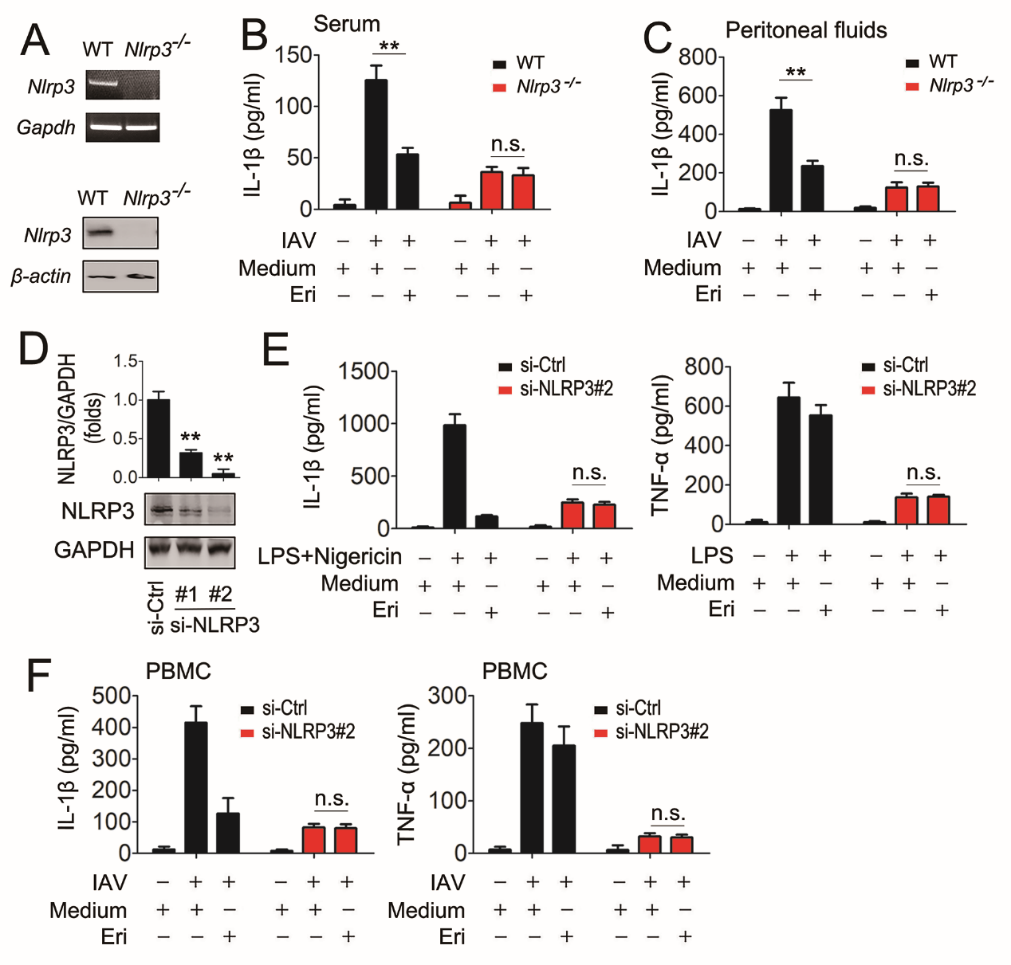


**Supporting Fig. 6. Eri inhibits IAV induced IL-1β production via NLRP3.**

(A) *Nlrp3* expression in the lung was detected using RT-PCR (upper panel) and western blot (lower panel) analyses (n = 3 for each group).

(B) WT or *Nlrp3^-/-^* mice were intranasally infected with 1×10^4^ pfu of WSN IAV, and were intraperitoneally injected with Eri (5 mg/kg) for 2 d. IL-1β protein levels in the bronchoalveolar lavage fluid (BALF) were determined by ELISA (n = 3 for each group).

(C) BMDMs from WT or *Nlrp3^-/-^* mice were infected WSN IAV (MOI=1) for 36 h, and treated with (5 nM) Eri for 3 h. IL-1β protein levels were determined by ELISA (n = 3 for each group).

(D) THP-1 cells were transfected with indicated si-RNAs for 48 h prior to real-time RT-PCR and western blot analyses.

(E) THP-1 cells were transfected with indicated si-RNAs for 42 h, and treated with LPS (1 µg/ml) for 3 h. Then, cells were treated with nigericin (2 μM) or Eri (5 nM) for 3 h. IL-1β (left panel) and TNF-α (right panel) protein levels were determined by ELISA.

(F) Freshly isolated PBMCs from C57BL/6J mice were transfected with indicated si-RNAs for 24 h and infected IAV for 12 h. Then, cells were treated with Eri (5 nM) for 3 h. IL-1β (left panel) and TNF-α (right panel) protein levels were determined by ELISA.

All experiments were repeated at least three times. Bar graphs present means ± SD, or means ± SEM (**P < 0.01; *P < 0.05, n.s., not significant)


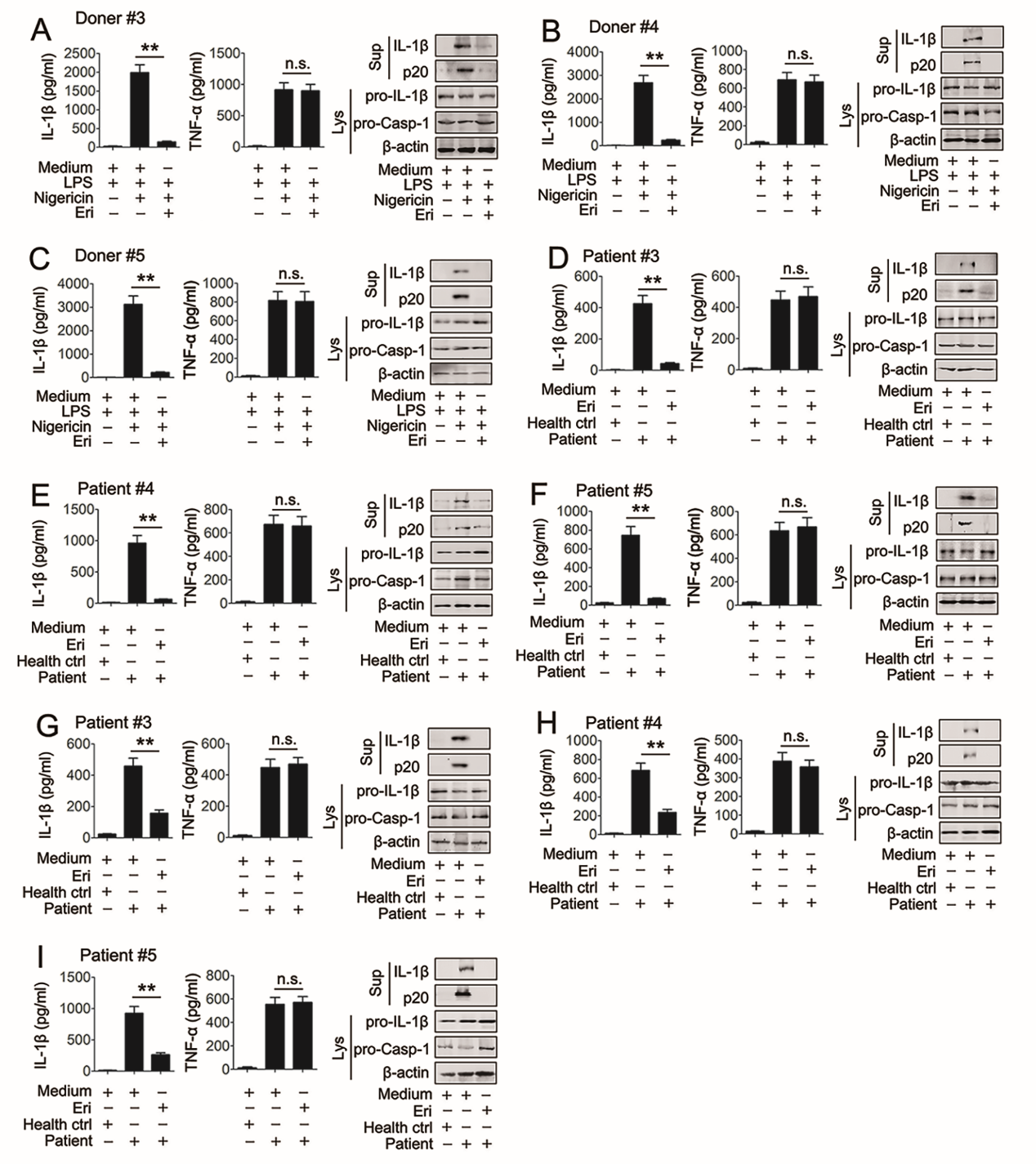


**Supporting Fig. 7. Eri is active for cells from healthy humans, IAV patients or gout patients, related to Fig. 7.**

(A-C) Experiments were performed as described in Fig. 7A, except PBMCs from donor #3 (A), donor #4 (B) and donor #5 (C) were used.

(D-F) Experiments were performed as described in Fig. 7C, except PBMCs from IAV patient #3 (A), IAV patient #4 (B) and IAV patient #5 (C) were used.

(G-I) Experiments were performed as described in Fig. 7E, except SFCs from gout patient #3 (A), gout patient #4 (B) and gout patient #5 (C) were used.

All experiments were repeated at least three times. Bar graphs present means ± SEM, n = 3 (**P < 0.01; *P < 0.05).


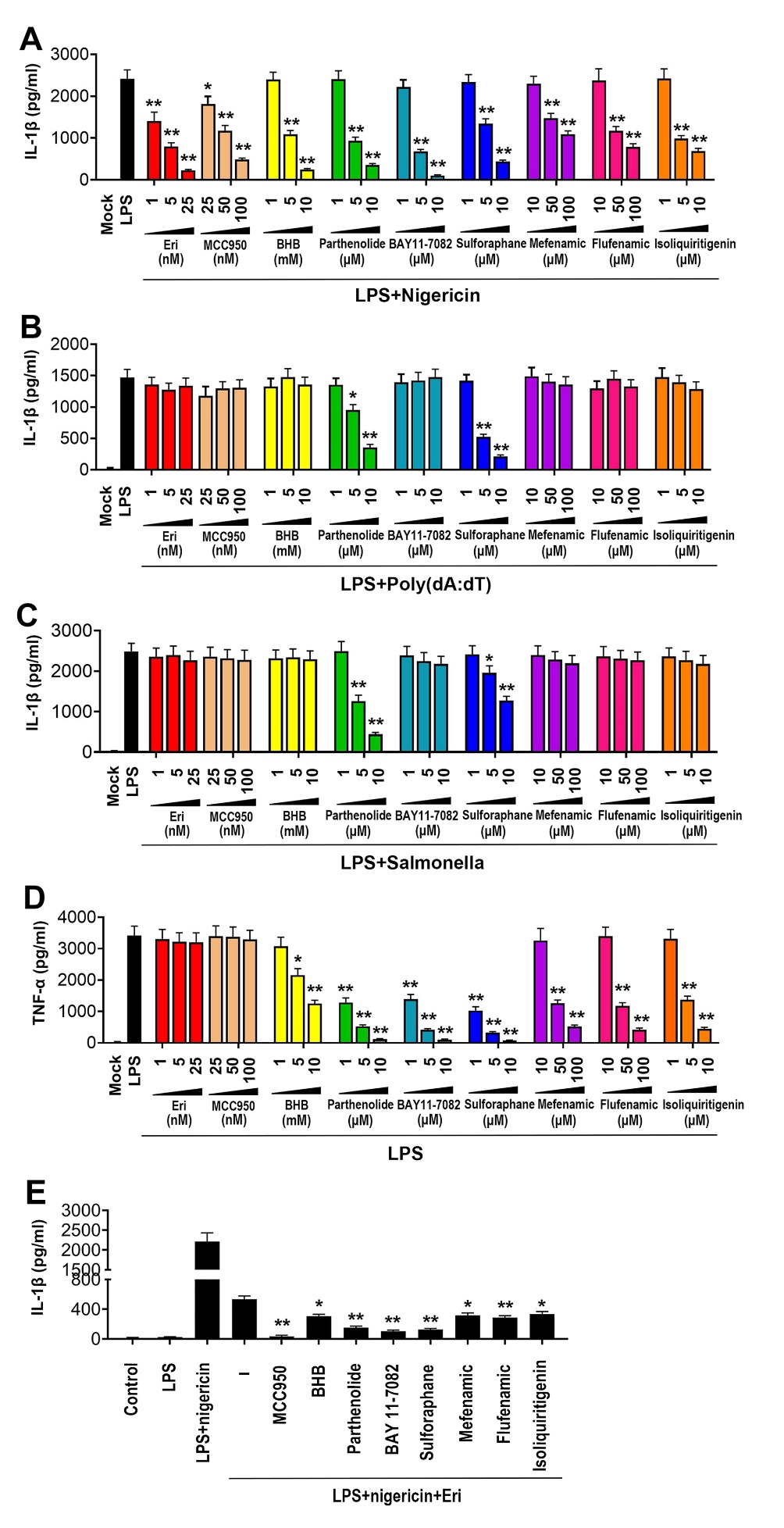


**Supporting Fig. 8. role of inhibitors on inflammasome activation.**

(A) BMDMs were treated with or without LPS (1 µg/ml) for 3 h, and then treated with nigericin (2 μM), Eri (5 nM) and the indicated inhibitors for 3 h. IL-1β protein levels were determined by ELISA.

(B) THP-1 cells were transfected with poly(dA:dT) (0.5 µg/ml) for 4 h, and then treated with Eri (5 nM), LPS (1 µg/ml) or the indicated inhibitors for 3 h. IL-1β protein levels were determined by ELISA.

(C) THP-1 cells were treated with *Salmonella typhimurium* (multiplicity of infection) or for 30 min, and then treated with Eri (5 nM), LPS (1 µg/ml) or the indicated inhibitors for 3 h. IL-1β protein levels were determined by ELISA.

(D) BMDMs were treated LPS (1 µg/ml) for 6 h, and then treated with Eri (5 nM) or the indicated inhibitors for 3 h. TNF-α protein levels were determined by ELISA.

(E) BMDMs were treated with or without LPS (1 µg/ml) for 3 h, and then treated with nigericin (2 μM), Eri (5 nM) and the indicated inhibitors for 3 h. IL-1β protein levels were determined by ELISA.

All experiments were repeated at least three times. Bar graphs present means ± SD (**P < 0.01; *P < 0.05, n.s., not significant).

**Table S1 Antibodies used in this study**

| Antibodies | Source | Dilution |
| --- | --- | --- |
| MEK7 | Abcam (ab52618) | 1:1000 |
| HA | Sigma (H6908) | 1:2000 |
| Flag | Sigma (M2) | 1:2000 |
| Myc | Abcam (ab32) | 1:2000 |
| ASC | Santa Cruz (sc-271054) | 1:500 |
| Caspase-1 | Cell Signaling (2225) | 1:1000 |
| β-actin | Abcam (ab179467) | 1:5000 |
| IL-1β | Cell Signaling (D3U3E) | 1:1000 |
| NLRP3 | Cell Signaling (D2P5E) | 1:1000 |
